# OPRM1 gene polymorphism linked to anxiety in cancer-related pain patients: an observational study

**DOI:** 10.3389/fpain.2026.1666510

**Published:** 2026-02-05

**Authors:** Jianwen Mo, Zhuolun Xie, Xiaolan Li, Lei Hu, Min Chen, Xiaolin Xia, Wei Wang

**Affiliations:** 1 Department of Oncology, Yunfu People’s Hospital, Yunfu, China; 2 Department of Radiation Oncology, Nanfang Hospital, Southern Medical University, Guangzhou, China; 3 Department of Pharmacy, Yunfu People's Hospital, Yunfu, China; 4 Guangdong Provincial Key Laboratory for Prevention and Control of Major Liver Disease, Southern Medical University Nanfang Hospital, Guangzhou, China

**Keywords:** cancer pain, numeric rating scale (NRS), OPRM1, Quality of Life Questionnaire Core 30 (QLQ-C30), self-rating anxiety scale (SAS), self-rating depression scale (SDS)

## Abstract

**Introduction:**

Anxiety is common in patients with cancer-related pain, affecting an increasing number of individuals annually and negatively impacting prognosis. Limited methods are available for early identification of anxiety in these patients. This study explores the link between the OPRM1 gene's polymorphism and anxiety in cancer patients.

**Methods:**

We prospectively recruited 76 patients experiencing pain from Yunfu People's Hospital and assessed them with questionnaires on anxiety, depression, quality of life, and other metrics before they commenced opioid treatment. Additionally, Numeric Rating Scale (NRS) scores and opioid usage data were recorded. In situ hybridization was used to analyze the OPRM1 polymorphism rs1799971. Based on the available data, appropriate opioid dosage conversion relationships were established.

**Results:**

This study found that 40.8% (31/76) exhibited anxiety symptoms. OPRM1 genotyping revealed 31.6% AA, 48.7% AG, and 19.7% GG genotypes, conforming to Hardy-Weinberg equilibrium. No genotype differences were found in morphine dosage or NRS pain scores. However, G allele carriers exhibited significantly lower anxiety scores (median 27.0 vs. 36.0, *p* = 0.017). Multivariate analysis identified G allele (OR = 0.15, *p* = 0.040) and antitumor therapy (OR = 0.10, *p* = 0.027) as protective against anxiety, while breakthrough pain (OR = 6.65), higher pretreatment NRS scores (OR = 2.80), and depression (OR = 61.03) were risk factors (all *p* < 0.05).

**Discussion:**

Our study indicates that G allele carriers exhibit lower anxiety levels, suggesting a certain correlation between the OPRM1 gene polymorphism and anxiety in cancer pain patients. Furthermore, there is a need to pay increased attention to the psychological health of cancer pain patients with the AA genotype.

## 1 Introduction

Among patients receiving cancer pain treatment, anxiety is highly prevalent, with approximately 10% of individuals experiencing varying degrees of anxiety, a number that is continually increasing annually (1–3). Studies indicate that anxiety can lead to a decline in overall quality of life (4). A meta-analysis demonstrated that anxiety in cancer pain patients adversely affects prognosis, with a 44% reduction in survival rates (5). Therefore, early identification of high-risk patients is crucial. However, current methods for early identification of anxious individuals among cancer pain patients remain limited.

Current early identification of anxiety primarily relies on factors such as age, disease stage, smoking status (6), reproductive phenotypes, including the age of menarche, age at first sexual intercourse (AFS) (7), and the availability of *μ*-opioid receptors (MOR) (8). The MOR, encoded by OPRM1, is widely distributed in the brain and plays key roles in pain processing, addiction, and emotional regulation. In the parabrachial nucleus (PBL), manipulating the activity of neurons expressing OPRM1 may trigger the occurrence of anxiety-like behaviors through the activation of ventral tegmental area (VTA) dopaminergic neurons, such as changes in respiratory frequency (9, 10). Therefore, the OPRM1 gene is considered to be a gene that influences anxiety and social behavior (11). However, it is currently unclear whether OPRM1 gene polymorphisms can be used for the early identification of cancer pain patients with comorbid anxiety.

During the treatment of cancer pain, it is particularly important to recognize and effectively manage symptoms of anxiety, as psychological health can improve the quality of life and the overall effectiveness of cancer pain treatment (12). We hypothesize that the OPRM1 gene could serve as early biomarkers for anxiety in cancer pain patients. To test this hypothesis, we conducted the present study. We prospectively recruited 76 patients with malignant tumors who were scheduled to receive opioid analgesic treatment for their pain and administered questionnaires assessing anxiety and other psychological factors upon admission. Patients were subsequently divided into two groups based on whether they carried the G allele of the OPRM1 gene. We then compared differences between the groups across various dimensions of the questionnaire scores. Ultimately, our study results confirmed that G allele carriers exhibited lower levels of anxiety. This insight paves the way for new, personalized psychological support treatments for cancer pain patients and promotes a more diversified approach to cancer care.

## 2 Methods

### 2.1 Study design and participants

This study is a prospective cohort study of the anxiety levels of cancer pain patients with different OPRM1 genotypes that are conducted at a single institution. Our study was approved by the Yunfu People's Hospital Medical Ethics Committee (2022A050) and was conducted in compliance with the Declaration of Helsinki and Ethical Guidelines for Medical and Health Research Involving Human Subjects. The corresponding authors designed, managed, and provided financial support for the study. All data collection occurred prospectively. Clinical data were registered by physicians. Data analysis was conducted by first authors under the guidance of a statistician. The eligibility criteria: (1) Participants aged over 18 years; (2) Cancer patients requiring appropriate opioid analgesics for pain relief; (3) Individuals with good compliance who can effectively return for follow-up visits; (4) non-cancer pain patients. Key exclusion criteria included: (1) Participants under 18 years of age; (2) Patients with non-cancer pain; (3) Patients suffering from severe mental disorders that might interfere with the normal conduct of the study; (4) Individuals with incomplete data; (5) Participants with poor compliance; (6) Individuals without complete and valid OPRM1 gene testing results. Informed consent was obtained from all individual participants included in the study.

### 2.2 Patient enrollment

**Figure 1:**
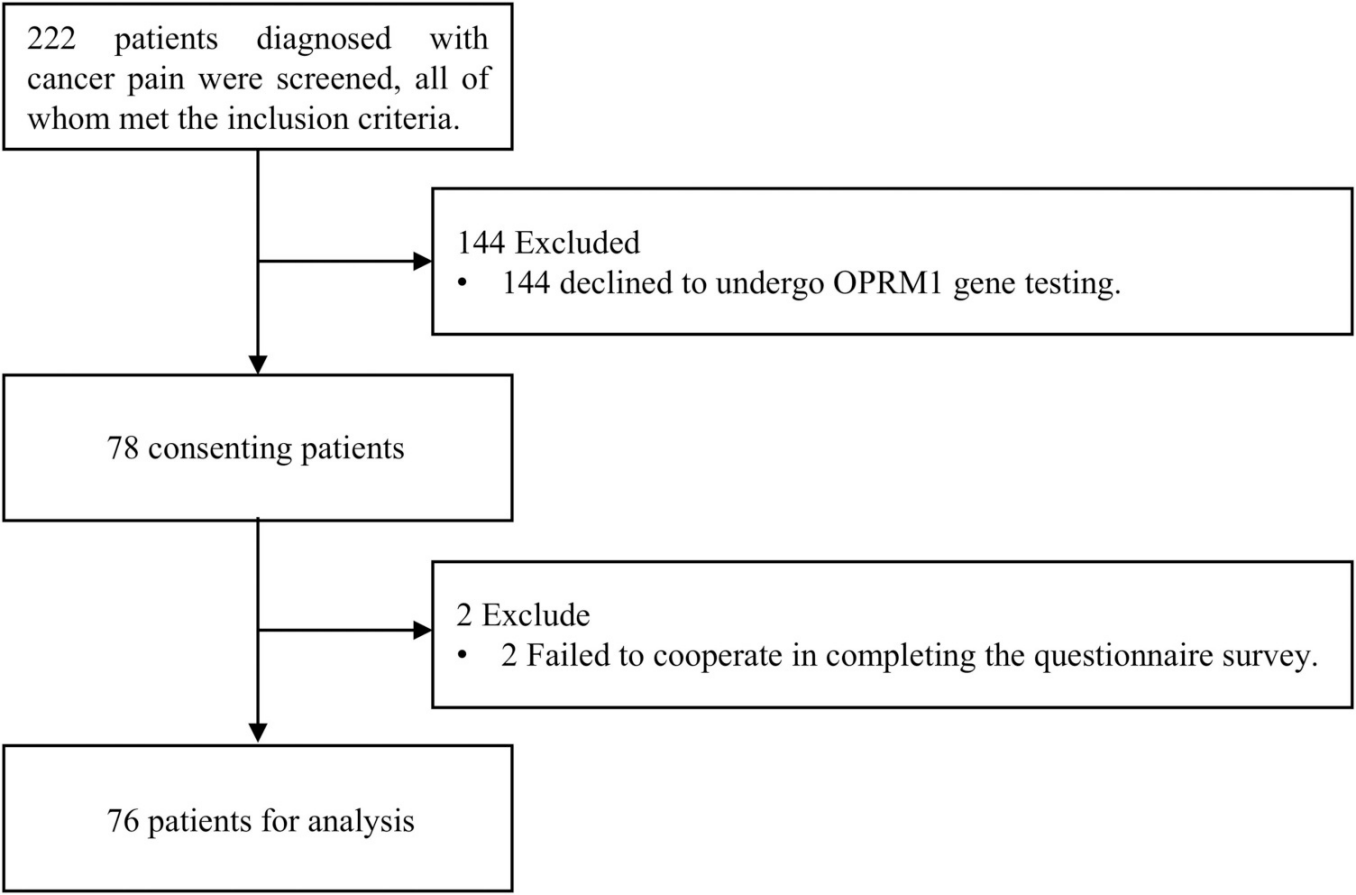
Study diagram of this study.

Prior to enrollment, all patients completed comprehensive pretreatment assessments to identify any opioid contraindications. Between January, 2023 to May, 2024, 76 cancer pain patients who met the inclusion criteria and were receiving opioid treatment participated in this study. All recruited patients were from Yunfu People's Hospital. The diagram is shown in (Figure 1).

### 2.3 Data collected

All patients completed relevant questionnaires before receiving opioid treatment.

Each patient's anxiety status was assessed using the Self-Rating Anxiety Scale (SAS). The total score was calculated by summing the ratings of all items. A total score above 36 points was considered a positive screen, indicating the possible presence of anxiety symptoms requiring further examination. The higher the score, the more severe the anxiety.

The European Organization for Research and Treatment of Cancer (EORTC) developed the Quality of Life Questionnaire Core 30 (QLQ-C30) to assess the quality of life in cancer pain patients (13). This questionnaire includes 30 items covering multiple domains of quality of life, such as overall health status, functional scales and symptom scales.

Each patient's depression status was evaluated using the Self-Rating Depression Scale (SDS). Items are rated on a 4-point scale (1–4 for negative statements, 4-1 for positive statements), with scores summed across all 20 items, multiplied by 1.25, and rounded to the nearest whole number to derive the standard total score. Scores below 50 indicate normal, 50-59 mild depression, 60-69 moderate depression, and 70-79 severe depression.

Nutritional status was assessed using a modified Subjective Global Assessment (SGA) with five grades: Normal (no weight loss, adequate intake), Mild malnutrition (5%–7.5% weight loss, slight dietary reduction), Moderate malnutrition (7.5%–10% weight loss, functional impairment), Severe malnutrition (>10% weight loss, marked depletion), and Overweight (BMI≥25 without malnutrition). Evaluations included weight history, dietary intake, and physical examination for muscle/fat loss.

All patients underwent pain assessment using the Numeric Rating Scale (NRS; 0–10 point scale) both before initiation and after completion of opioid therapy to evaluate treatment-related changes in pain status. All patients underwent an NRS assessment for pain before receiving opioid treatment. After administering an equivalent dose of opioids and once the patients experienced pain relief, another NRS assessment was conducted. The dose of opioid medication required for pain relief was recorded for each patient.

### 2.4 OPRM1 genotyping

Oral mucosal cell samples were collected from the buccal walls of all participants using disposable sampling swabs and reserved for subsequent use. Swabs that could not be analyzed immediately should be stored at 2–8 °C and should not exceed 7 days in storage. After collecting, the swabs were allowed to thaw in the reagent room, followed by instantaneous centrifugation and reserved for further processing. A volume of 400 μL of sample extraction solution (CQ-ENH type) was pipetted into a 1.5 mL centrifuge tube, into which the swab head was immersed, and the stem of the swab was broken off so that the swab head was submerged in the extraction solution. The mixture was vortexed to ensure homogeneity (allowed to stand for 1 min). Subsequently, 1.0 μL of the processed sample was spotted onto the wall of the reagent tube, followed by centrifugation before being loaded onto the detection instrument. According to the manufacturer's instructions, *in situ* hybridization (ISH) was used to analyze the OPRM1 rs1799971 (A118G) gene polymorphism (reagents provided by Jinan Guangyin Medical Technology Co., Ltd.). The FISH method employed in this study is a liquid-phase hybridization system specifically designed for SNP genotyping of extracted DNA, rather than for traditional tissue localization applications. This method has undergone consistency validation using blood and oral exfoliated cell samples, with validation results demonstrating 100% genotypic concordance. The method is reliable and suitable for genotyping analysis of oral swab samples. Full validation data are provided in Attachment 1.

### 2.5 Medications

In the course of our study, various types of opioid medications were utilized, making it necessary to convert to equivalent doses when comparing patients to different medications. We conducted a comparative analysis of the equivalent analgesic doses of several common analgesics and have listed the dose conversion ratios for these drugs administered via intramuscular (IM) and oral (PO) routes in. Based on the total daily oral morphine consumption, we converted to the recommended dosage of fentanyl patches. These dose conversion relationships are derived from publications by the American Society of Health-System Pharmacists (ASHP) and the instructions for use accompanying the fentanyl transdermal patch (Duragesic) (38).

### 2.6 Outcome

The primary objective of this study is to investigate the potential association between the OPRM1 gene and anxiety in a cohort of cancer pain patients. Secondary objectives include examining the correlation between the OPRM1 gene and the dose of opioid medication used.

### 2.7 Statistical analyses

Quantitative data are presented as median (interquartile range) or mean ± standard deviation, and categorical data are presented as number of cases (*n*, %). No missing data was encountered in the dataset utilized for this analysis. The relative frequencies of each genotype and allele were calculated and compared using chi-square analysis. Statistical comparisons of SAS, SDS, and QLQ-C30 scores among chronic cancer pain patients were performed using the “CreateTableOne()” function from the “tableone” R package, which automatically selects appropriate tests: *t*-tests or Wilcoxon rank-sum tests for continuous variables based on normality distribution, and Pearson's chi-square tests (with automatic switch to Fisher's exact test when expected cell counts <5) for categorical variables. To investigate factors associated with anxiety in cancer patients with pain, logistic regression analysis was employed. Variables with a significance level of *p* < 0.05 were included in the subsequent multivariate analysis. For categorical variables with multiple levels, the entire variable was retained in multivariate analysis if any level reached statistical significance (*p* < 0.05), maintaining the integrity of the categorical structure. Multicollinearity among independent variables was evaluated using variance inflation factors (VIFs). Model fit was assessed using a likelihood ratio test.

A *P*-value < 0.05 was considered statistically significant. Statistical analyses were carried out with R software package version 4.4.0 and SPSS 27.0.

## 3 Results

### 3.1 Patients

The main clinical and demographic characteristics of patients are presented in (Table 1).

**Table 1:** Patient baseline characteristics table.

Patient Characteristics (*n* = 76)		Value
Gender, *n* (%)	Male	61 (80.26)
	Female	15 (19.74)
Age (years)	Median [IQR]	64 [54, 70]
Height (m)		1.65 [1.60, 1.68]
Weight (Kg)		54.00 [48.62, 57.25]
BMI (Kg/m^2^)		20.07 [18.12, 22.05]
Smoking history, *n* (%)	YES	18 (23.68)
	NO	58 (76.32)
History of drug abuse, *n* (%)	YES	0 (0.00)
	NO	76 (100.00)
Cancer type, *n* (%)	Head and Neck	20 (26.32)
	Lung	15 (19.74)
	Colon	11 (14.47)
	Liver	8 (10.53)
	Esophagus	4 (5.26)
	Stomach	3 (3.95)
	Prostate	3 (3.95)
	Others^1^	10 (13.16)
Pain location, *n* (%)	Abdomen	24 (31.58)
	Chest	14 (18.42)
	Head	21 (27.63)
	Limbs	6 (7.89)
	Waist	3 (3.95)
	Multiple Sites^2^	5 (6.58)
	Others^3^	3 (3.95)
Antitumor therapy, *n* (%)	YES	56 (73.68)
	NO	20 (26.32)
Opioids, *n* (%)	Oxycodone	53 (69.74)
	Tramadol	8 (10.53%)
	Fentanyl	6 (7.89%)
	Morphine	5 (6.58%)
	combinded^4^	4 (5.26)
Breakthrough pain, *n* (%)	YES	23 (30.26)
	NO	53 (69.74)
Depression, *n* (%)	Normal	48 (63.16)
	Mild	17 (22.37)
	Moderate	9 (11.84)
	Severe	2 (2.63)
Subjective Global Assessment (SGA), *n* (%)	Normal	20 (26.32)
	Mild malnutrition	21 (27.63)
	Moderate malnutrition	10 (13.16)
	Severe malnutrition	20 (26.32)
	Overweight	5 (6.58)
Anxiety, *n* (%)	Yes	31 (40.79)
	No	45 (59.21)
Allele frequency		A:0.560 G:0.440

IQR, interquartile range; BMI, body mass index; ^1^-Including Bladder Cancer, Adrenal Sarcoma, Cervical Cancer; ^2^-Pain in Two or More Sites; ^3^-Including Anal, Foot, Liver Areas, etc.; ^4^-Use of Two or More Opioids.

The population was predominantly male (80.26%) with a median age of 64 years (IQR:54–70). Anthropometric measurements revealed a median height of 1.65 m, weight of 54.00 kg, and BMI of 20.07 kg/m^2^. Smoking history was reported in 23.68% of patients, while no cases of drug abuse were documented. Regarding cancer distribution showed head and neck tumors as most prevalent (26.32%), followed by lung (19.74%) and colon cancers (14.47%). Pain locations were primarily abdominal (31.58%) and cephalic (27.63%). Current antitumor therapy was administered to 73.68% of patients. The psychological evaluation revealed that 31 patients (40.79%) presented with anxiety. Regarding depressive symptoms, 17 cases (22.37%) were classified as mild, 9 (11.84%) as moderate, and 2 (2.63%) as severe. Nutritional status evaluation demonstrated comparable proportions of normal nutrition (26.32%) and severe malnutrition (26.32%). The cohort exhibited allele frequencies of 0.560 (A) and 0.440 (G) for the studied genetic variant.

### 3.2 OPRM1 genotype and opioid correlation

The morphine dosage required by OPRM1 genotype patients and their NRS scores are presented in (Table 2). A comparative analysis of equianalgesic dosing was conducted, with intramuscular and oral dose conversion ratios provided in Supplementary Table S1 and fentanyl patch dosing recommendations based on daily oral morphine intake summarized in Supplementary Table S2.

**Table 2:** Morphine-equivalent opioid dosage requirements and numerical rating scale (NRS) pain scores stratified by genotype.

Index	Allele frequency				Test statistic^1^	*p*-value
	A/A *n* = 24 (31.6%)	A/G *n* = 37 (48.7%)	G/G *n* = 15 (19.7%)	A/G + G/G *n* = 52 (68.4%)		
Dose of Morphine (mg/day) (SD)	52.73 (39.01)	50.51 (51.82)	50.73 (56.18)	50.58 (52.56)	0.538	0.911
NRS score before treatment (0–10) (SD)	4.38 (0.98)	4.22 (1.13)	4.00 (1.00)	4.15 (1.09)	2.931	0.402
NRS score after treatment (0–10) (SD)	1.75 (0.42)	1.74 (0.48)	1.70 (0.53)	1.73 (0.49)	0.032	0.999
*Δ*NRS^2^	2.62 (1.1)	2.47 (0.98)	2.30 (0.88)	2.42 (0.95)	0.690	0.875
Average Dose (mg/day) (SD)	21.64 (23.81)	22.60 (24.85)	21.09 (13.85)	21.45 (17.68)	0.512	0.916

SD, standard deviation; NRS, Numerical Rating Scale; ^1^- Kruskal–Wallis H Test was used to examine; ^2^- decrease in Numerical Rating Scale.

In this study, the OPRM1 genotyping of 76 pain patients revealed 24 cases (31.6%) with the AA genotype, 37 cases (48.7%) with the AG genotype, and 15 cases (19.7%) with the GG genotype. Statistical analysis confirmed that the genotype distribution was in accordance with Hardy-Weinberg equilibrium, indicating that the sample population was representative. We initially analyzed whether there were differences in pain and opioid dosage requirements among cancer pain patients with different genotypes. The distribution of opioids used in this population is as follows: oxycodone (*n* = 53, 69.74%), tramadol (*n* = 8, 10.53%), fentanyl (*n* = 6, 7.89%), morphine (*n* = 5, 6.58%), and combination therapy (*n* = 4, 5.26%). The results showed no statistically significant differences among the different genotype groups in terms of daily morphine dosage (*p* = 0.911), NRS scores before and after treatment (*p* = 0.402, *p* = 0.999), *Δ*NRS scores (*p* = 0.875), and the Average Dose of morphine (*p* = 0.916).

### 3.3 Patients carrying at least one G allele had significantly lower anxiety scores

The scores for each dimension and the statistical results are presented in (Table 3).

**Table 3:** Self-Rating anxiety scale (SAS), self-rating depression scale (SDS) and quality of life questionnaire core 30 (QLQ-C30) score.

Index	A/A median [IQR]	A/G + G/G median [IQR]	*p*-value
Global health status/QoL (GQOL)	3.00 [2.00, 3.00]	3.00 [2.00, 4.00]	0.095
Physical functioning (PF)	2.10 [1.55, 3.05]	2.00 [1.55, 2.60]	0.682
Role functioning (RF)	2.25 [1.88, 3.62]	2.00 [1.50, 3.00]	0.578
Social functioning (SF)	2.00 [1.38, 3.00]	2.00 [1.88, 2.25]	0.929
Emotional functioning (EF)	1.75 [1.19, 2.00]	1.25 [1.00, 1.75]	0.070
Cognitive functioning (CF)	1.75 [1.00, 2.12]	1.50 [1.00, 2.00]	0.412
Pain (PA)	2.00 [1.50, 2.50]	2.00 [1.50, 2.50]	0.94
Fatigue (FA)	2.00 [1.58, 2.75]	2.00 [1.58, 2.33]	0.713
Nausea and vomiting (NV)	1.00 [1.00, 2.00]	1.00 [1.00, 2.00]	0.624
Dyspnea (DY)	1.00 [1.00, 2.00]	1.00 [1.00, 2.00]	0.995
Insomnia (SL)	2.00 [1.00, 2.25]	2.00 [1.00, 2.00]	0.995
Appetite loss (AP)	2.00 [1.75, 2.25]	2.00 [1.00, 2.00]	0.811
Constipation (CO)	2.00 [1.00, 2.00]	1.00 [1.00, 2.00]	0.185
Diarrhea (DI)	1.00 [1.00, 1.00]	1.00 [1.00, 1.00]	0.944
Financial difficulties (FI)	2.00 [2.00, 2.25]	2.00 [2.00, 2.00]	0.751
SDS Score	42.00 [32.75, 58.25]	37.00 [28.75, 51.25]	0.216
SAS Score	36.00 [28.00, 42.00]	27.00 [22.00, 36.25]	0.016

*P* < 0.05, statistically significant; SD = standard deviation; SDS, the total score ranges from 20 to 80 points; SAS, the total score ranges from 20 to 80 points; QLQ-C30, Except for the GQOL total score, which is out of 6 points, all others are out of 3 points.

The study results indicate that there are no significant differences between patients with the A/A genotype and the combined G allele carriers in multiple dimensions of the QLQ-C30 and the SDS scores. However, a significant difference was observed in anxiety scores between genotype groups (*p* = 0.017). Patients with the A/A genotype demonstrated significantly higher median anxiety scores (36.00 [IQR: 28.00–42.00) compared to those with A/G + G/G genotypes (27.00 [IQR: 22.00–36.25).

### 3.4 Genotype has a significant impact on anxiety status

To identify key determinants of anxiety status, univariate analysis revealed significant associations with genotype, pain location, antitumor therapy, breakthrough pain, pretreatment NRS scores, and depression. Notably, multivariate analysis confirmed that G allele carriers (OR:0.15, 95%CI: 0.02–0.91, *P* = 0.040) and antitumor therapy (OR:0.10, 95%CI: 0.01–0.77, *P* = 0.027) served as independent protective factors, while breakthrough pain (OR:6.65, 95%CI: 1.00–44.16, *P* = 0.050), higher pretreatment NRS scores (OR:2.80, 95%CI: 1.05–7.44, *P* = 0.039), and depression (OR:61.03, 95%CI: 8.40–443.66, *P* < 0.001) emerged as independent risk factors for anxiety (Table 4). Most VIFs were below 5, with the exception of “AST after treatment” which had a VIF of 6.25 (Supplementary Table S3). The full model provided a significantly better fit to the data than the null model, as evidenced by a likelihood ratio test (*χ*^2^= 75.33, *p* < 0.001).

**Table 4:** Univariate and multivariate analysis of anxiety Status.

Variable		Univariate analysis		Multivariate analysis	
		OR (95%Cl)	*P*-value	OR (95%Cl)	*P*-value
Gene	AA	1 [Reference]		1 [Reference]	
	AG + GG	0.35 (0.13–0.94)	0.037	0.15 (0.02–0.91)	0.040
Age, years		0.99 (0.95–1.03)	0.575		
Sex	Female	1 [Reference]			
	Male	1.04 (0.33–3.30)	0.945		
Smoking history	NO	1 [Reference]			
	YES	1.64 (0.56–4.75)	0.365		
Diagnosis	Chest	1 [Reference]			
	Abdomen	1.21 (0.31–4.73)	0.782		
	Head	0.30 (0.08–1.14)	0.078		
	Abdomenr	0.55 (0.10–2.89)	0.476		
	Others	0.40 (0.09–1.73)	0.223		
Pain loction	Abdomen	1 [Reference]		1 [Reference]	
	Chest	0.40 (0.10–1.55)	0.183	0.46 (0.05–3.98)	0.482
	Head	0.22 (0.06–0.81)	0.023	0.31 (0.04–2.39)	0.263
	Others	0.50 (0.14–1.77)	0.281	0.15 (0.01–1.54)	0.109
Antitumor therapy	NO	1 [Reference]		1 [Reference]	
	YES	0.26 (0.09–0.75)	0.013	0.10 (0.01–0.77)	0.027
Breakthrough pain	NO	1 [Reference]		1 [Reference]	
	YES	3.29 (1.19–9.11)	0.022	6.65 (1.00–44.16)	0.050
Opioid Dosage, mg/day		1.01 (1.00–1.02)	0.174		
NRS score before treatment		1.91 (1.15–3.18)	0.013	2.80 (1.05–7.44)	0.039
NRS score after treatment		1.18 (0.44–3.20)	0.740		
AST before treatment		1.01 (0.99–1.03)	0.486		
ALT before treatment		1.01 (1.00–1.01	0.256		
AST after treatment		1.01 (0.99–1.03)	0.316		
ALT after treatment		1.01 (1.00–1.02)	0.197		
Depression	NO	1 [Reference]		1 [Reference]	
	YES	23.00 (6.73–78.64)	<0.001	61.03 (8.40–443.66)	<0.001
Subjective Global Assessment (SGA)	Normal	1 [Reference]			
	Malnutrition	0.49 (0.17–1.39)	0.177		
	Overweight	0.20 (0.02–2.17)	0.188		

OR = Odds Ratio; 95% CI = 95% confidence interval; *P* < 0.05, statistically significant.

## 4 Discussion

In this small-scale cohort study, we prospectively enrolled 76 cancer patients with varying OPRM1 genotypes. Of these, 40.79% (*n* = 31) exhibited anxiety symptoms, with AA genotype carriers demonstrating significantly higher susceptibility. Both univariate and multivariate analyses identified AA genotype as an independent risk factor for anxiety. These findings suggest an association between the OPRM1 AA genotype and anxiety development in cancer pain patients, supporting its potential utility as a biomarker for early psychological intervention in this population.

No significant differences in pain scores or morphine use were found across OPRM1 genotypes. High baseline pain was an independent risk factor for anxiety, suggesting pain itself does not mediate genotype-related anxiety. Additionally, anxiety was significantly associated with OPRM1 genotype, antitumor therapy, breakthrough pain, and depression. These factors may potentially exacerbate anxiety symptoms (14, 15).

Chen et al. reported fewer female pain patients with anxiety or depression carried the GG genotype compared to the AA/AG genotype, consistent with our findings (16). Although no significant association was found between OPRM1 G allele and depression, G allele carriers showed lower depression scores. Numerous studies have demonstrated that anxiety negatively impacts pain treatment outcomes by increasing pain sensitivity (17) and causing muscle tension (18), which exacerbates pain (19–21). A study on how psychological factors affect postoperative pain has indicated that among many factors, state anxiety has the most significant impact on pain, followed by negative suggestions from other patients and the patient's own level of attention to pain (22). Unlike depression, anxiety shows a strong correlation with pain intensity and is more disruptive to daily functioning in advanced cancer patients (23, 24). This may explain why no significant differences in levels of depression have been observed among patients with chronic cancer-related pain, while anxiety is one of the key factors affecting pain management in such patients.Although numerous studies have suggested that the G allele may increase the dosage of opioids required (25–28), our research on cancer pain patients observed a different situation. However, this finding exhibit population heterogeneity. The association is more commonly observed in Asian populations (28, 29). While most studies on postoperative pain patients report this correlation, numerous studies involving cancer pain patients have failed to demonstrate such an association (30–32). A study revealed that G allele carriers exhibit an overall reduction in baseline μ-opioid receptor availability in brain regions associated with pain processing and emotional regulation (33). Furthermore, neuroimaging data demonstrated that among chronic pain patients (e.g., fibromyalgia), G allele carriers showed weakened functional connectivity within the prefrontal regulatory network, coupled with enhanced activation in the posterior cingulate cortex (PCC), despite displaying comparable pain ratings to AA homozygotes (34). This suggests distinct pain modulation processes may exist across OPRM1 genotypes. These findings potentially elucidate the key mechanism underlying the observed association between genotype and anxiety in this study, while no direct correlation was found with pain intensity or analgesic requirements. Future investigations should further explore the specific effects of this genotype on the aforementioned emotion-regulation circuits within the context of cancer-related pain.

Our study has several limitations that should be considered. First, the modest sample size (*N* = 76) resulted in small subgroup populations for each genotype, which may have reduced statistical power. Future research should validate these findings in larger multicenter cohorts and examine the longitudinal psychological outcomes associated with OPRM1 genotypes. Second, the significant gender imbalance, with males comprising 80% of the cohort, may restrict the generalizability of the results to female populations. Third, the heterogeneity in cancer types included in this study could introduce tumor-specific confounding effects that were not fully accounted for in the analysis.

## 5 Conclusions

Our study indicates that there is a heightened need to focus on the psychological well-being of patients with the A/A genotype. It is imperative to recognize the inseparable relationship between anxiety and pain, where pain can induce anxiety, and anxiety, in turn, can exacerbate pain, creating a vicious cycle. Consequently, the concurrent management of pain and psychological health is essential for achieving better prognostic outcomes. Non-pharmacological interventions, such as hypnosis, acupuncture, massage therapy, aromatherapy, music therapy, and enhanced nursing care, can effectively alleviate pain, ameliorate anxiety and emotional fluctuations, and enhance the quality of life (35–37). Future research should validate these findings in larger multicenter cohorts and examine the longitudinal psychological outcomes associated with OPRM1 genotypes.

## Data Availability

The raw data supporting the conclusions of this article will be made available by the authors, without undue reservation.
