# Supplementary material for: OPRM1 gene polymorphism linked to anxiety in cancer-related pain patients: an observational study
Source: Front Pain Res (Lausanne). 2026 Feb 5;7:1666510. doi: 10.3389/fpain.2026.1666510 (PMC12916677; doi:10.3389/fpain.2026.1666510)
Supplement: Supplementary file 1 [file Table1.docx]

TABLE S1 Dose Conversion Relationships for Different Opioids

| Drug Name | Equivalent Analgesic Dose (mg) | |
| --- | --- | --- |
|  | IM^1^ | PO^2^ |
| Morphine | 10 | 30(If Repeated Dosing) |
| Methadone | 10 | 20 |
| Oxycodone | 15 | 30 |
| Tramadol | 100 | 120 |

^1^- Intramuscular; ^2^- Per Os (which means "by mouth" in Latin)
