# Supplementary material for: OPRM1 gene polymorphism linked to anxiety in cancer-related pain patients: an observational study
Source: Front Pain Res (Lausanne). 2026 Feb 5;7:1666510. doi: 10.3389/fpain.2026.1666510 (PMC12916677; doi:10.3389/fpain.2026.1666510)
Supplement: Supplementary file 2 [file Table2.docx]

TABLE S2 Recommended Fentanyl Patch Dosage Based on Daily Oral Morphine Dose

| 24-Hour Oral Morphine Dose (mg/24h) | Fentanyl Release Rate ( μg/h) | Fentanyl Patch Content (mg) |
| --- | --- | --- |
| <135(Adult Patients) | 25 | 4.125 |
| 135-224 | 50 | 8.250 |
| 225-314 | 75 | 12.375 |
