# Supplementary material for: OPRM1 gene polymorphism linked to anxiety in cancer-related pain patients: an observational study
Source: Front Pain Res (Lausanne). 2026 Feb 5;7:1666510. doi: 10.3389/fpain.2026.1666510 (PMC12916677; doi:10.3389/fpain.2026.1666510)
Supplement: Supplementary file 3 [file Table3.docx]

TABLE S3 Multicollinearity Diagnostics

| Variable | VIF Value |
| --- | --- |
| Gene | 2.86 |
| Age | 3.91 |
| Sex | 2.67 |
| Smoking history | 2.30 |
| Diagnosis | 2.05 |
| Pain loction | 2.59 |
| Antitumor therapy | 3.25 |
| Breakthrough pain | 1.50 |
| Opioid Dosage | 2.38 |
| NRS score before treatment | 1.93 |
| NRS score after treatment | 1.51 |
| AST before treatment | 4.81 |
| ALT before treatment | 3.69 |
| AST after treatment | 6.25 |
| ALT after treatment | 4.37 |
| Depression | 4.70 |
| SGA | 1.54 |

VIF: Variance Inflation Factors.
