# Supplementary material for: OPRM1 gene polymorphism linked to anxiety in cancer-related pain patients: an observational study
Source: Front Pain Res (Lausanne). 2026 Feb 5;7:1666510. doi: 10.3389/fpain.2026.1666510 (PMC12916677; doi:10.3389/fpain.2026.1666510)
Supplement: Supplementary file 4 [file Datasheet1.pdf]

Comparative Verification Report of Blood and Oral Exfoliated Cells

I. Purpose of Verification

To verify the consistency of test results between blood samples and oral exfoliated cell samples, demonstrating that both blood samples and oral exfoliated cell samples are suitable for fluorescence in situ hybridization (FISH). The verification methods and report are as follows:

II. Verification Content and Methods

1. Subjects

1.1 Sequencing reaction universal kit (Luji Medical Equipment No. 20180020)

1.2 Sample extraction solution (Luji Medical Equipment No. 20180049) 1.3 Samples: EDTA-anticoagulated peripheral blood samples and oral swabs from 3 individuals (the same 3 individuals), regardless of disease type, testing purpose, gender, or age.

1.4 Detection loci: MTHFR 068rs1801133 (C>T); ALDH2 020rs671 (G>A); APOE 316rs429358 (T>C); APOE 312rs7412 (C>T); PAI-1 027rs1799762 (4G/5G); MTRR 040rs1801394 (A>G)

2. Content and Methods

The blood and oral swab samples were processed separately using the sample extraction solution to obtain leukocyte samples and oral exfoliated cell samples. The sequencing reaction universal kit from Guangyin Medical Technology Co., Ltd. was used for on-machine testing to evaluate the following performance:

Result consistency: The processed leukocyte samples from blood and oral exfoliated cell samples were added to the 6-locus reagent separately and run simultaneously on the machine. After the experiment, the results of the two sample types were compared, demonstrating 100% consistency.

3. Experimental Requirements

3.1 The laboratory must strictly adhere to the partitioning requirements of the Clinical Testing Center to ensure standardized operations and accurate, reliable results.

3.2 The operators should be familiar with the methodological principles and procedures, capable of proper sample handling, and able to smoothly perform leukocyte extraction, sample loading, and machine operation according to the workflow.

3.3 Before the experiment, the laboratory must establish the experimental testing conditions in accordance with the instructions provided with the reagent under evaluation.

4. Operating Procedures

4.1 Testing Process: EDTA anticoagulation tube, oral swab tube → specimen acceptance → pre-test preparation → pre-processing → sample loading → on-machine testing → result output.

4.2 Testing steps must strictly comply with the relevant standard operating procedures (SOP).

III. Verification Results

1. Consistency: Results are shown in the table below.

Well Number	Well Position	Locus Name	Test Result	Sample Number	Medical Record Number	Submission Date
0	A1	068rs1801133(C>T)	CT	1	blood	2023-01-10
1	A2	020rs671(G>A)	AG	1	blood	2023-01-10
2	A3	316rs429358(T>C)	TT	1	blood	2023-01-10
3	A4	312rs7412(C>T)	CC	1	blood	2023-01-10
4	A5	027rs1799762(4G/5G)	4G4G	1	blood	2023-01-10
5	A6	040rs1801394(A>G)	AG	1	blood	2023-01-10
6	B1	068rs1801133(C>T)	CC	2	blood	2023-01-10
7	B2	020rs671(G>A)	AG	2	blood	2023-01-10
8	B3	316rs429358(T>C)	TT	2	blood	2023-01-10
9	B4	312rs7412(C>T)	CC	2	blood	2023-01-10
10	B5	027rs1799762(4G/5G)	5G5G	2	blood	2023-01-10
11	B6	040rs1801394(A>G)	AG	2	blood	2023-01-10
12	C1	068rs1801133(C>T)	CT	3	blood	2023-01-10
13	C2	020rs671(G>A)	GG	3	blood	2023-01-10
14	C3	316rs429358(T>C)	TT	3	blood	2023-01-10
15	C4	312rs7412(C>T)	CC	3	blood	2023-01-10
16	C5	027rs1799762(4G/5G)	4G5G	3	blood	2023-01-10
17	C6	040rs1801394(A>G)	AG	3	blood	2023-01-10
18	D1					2023-01-10
19	D2					2023-01-10
20	D3					2023-01-10
21	D4					2023-01-10
22	D5					2023-01-10
23	D6					2023-01-10
24	E1	068rs1801133(C>T)	CT	1	oral	2023-01-10
25	E2	020rs671(G>A)	AG	1	oral	2023-01-10
26	E3	316rs429358(T>C)	TT	1	oral	2023-01-10
27	E4	312rs7412(C>T)	CC	1	oral	2023-01-10
28	E5	027rs1799762(4G/5G)	4G4G	1	oral	2023-01-10
29	E6	040rs1801394(A>G)	AG	1	oral	2023-01-10
30	F1	068rs1801133(C>T)	CC	2	oral	2023-01-10
31	F2	020rs671(G>A)	AG	2	oral	2023-01-10
32	F3	316rs429358(T>C)	TT	2	oral	2023-01-10

33	F4	312rs7412(C>T)	CC	2	oral	2023-01-10
34	F5	027rs1799762(4G/5G)	5G5G	2	oral	2023-01-10
35	F6	040rs1801394(A>G)	AG	2	oral	2023-01-10
36	G1	068rs1801133(C>T)	CT	3	oral	2023-01-10
37	G2	020rs671(G>A)	GG	3	oral	2023-01-10
38	G3	316rs429358(T>C)	TT	3	oral	2023-01-10
39	G4	312rs7412(C>T)	CC	3	oral	2023-01-10
40	G5	027rs1799762(4G/5G)	4G5G	3	oral	2023-01-10
41	G6	040rs1801394(A>G)	AG	3	oral	2023-01-10

2. Original result screenshots are as follows:

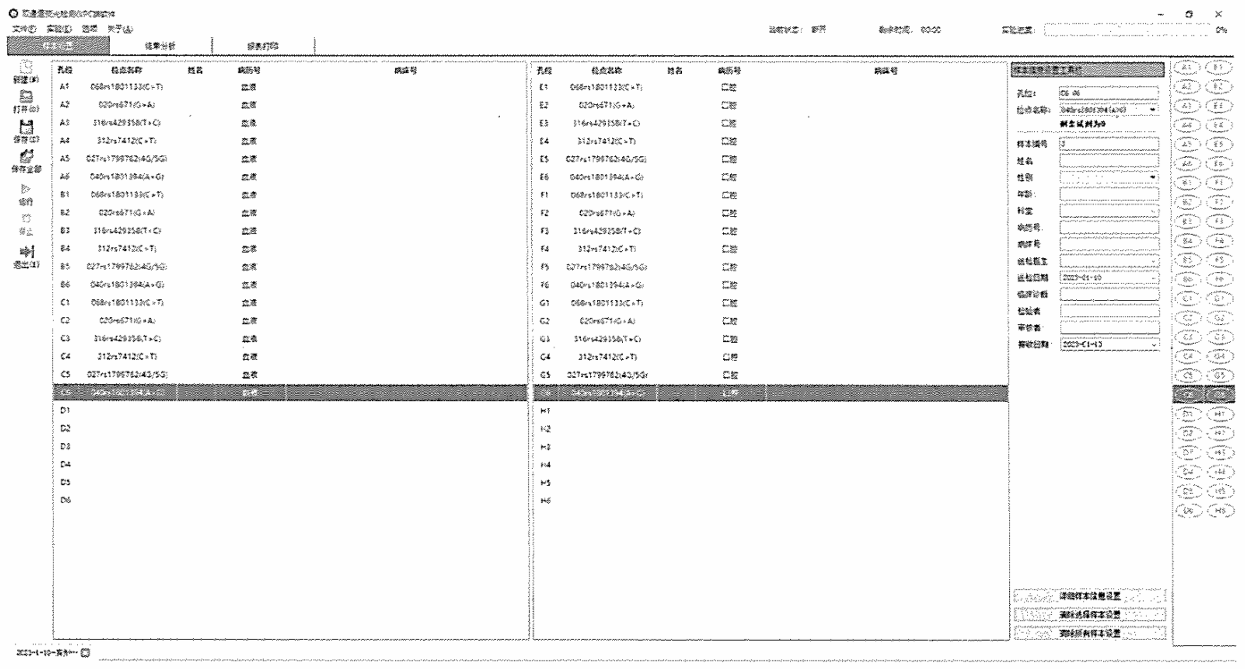

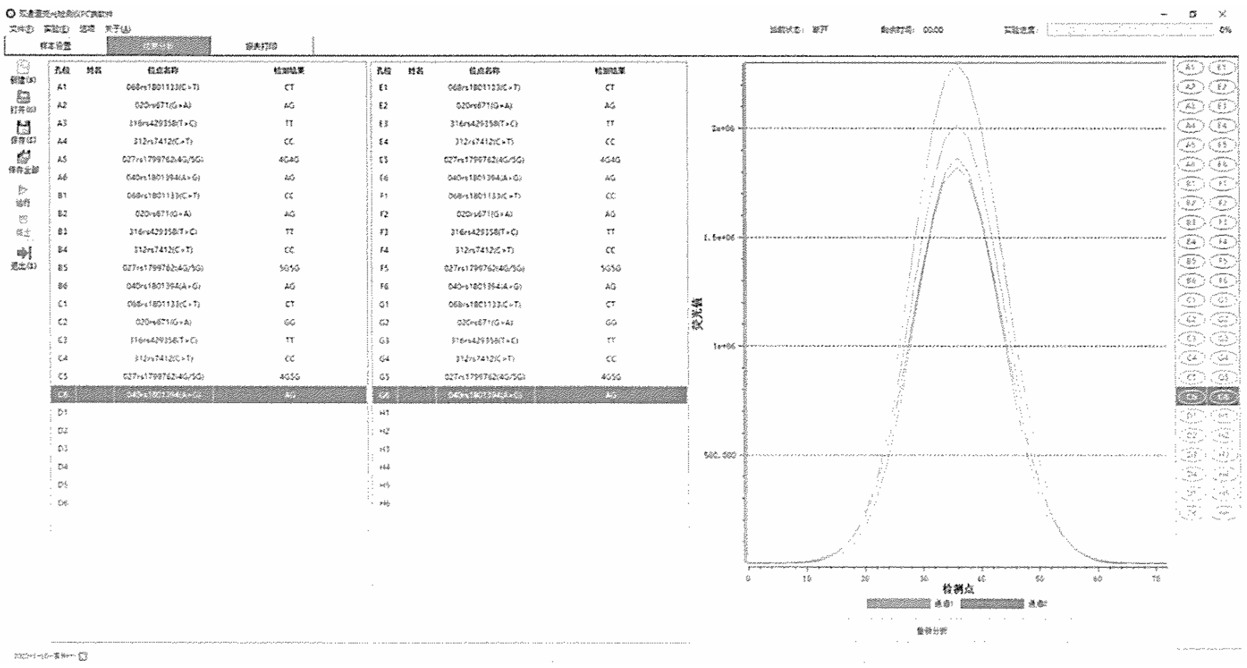

IV. Test Conclusion

This experiment demonstrated 100% consistency between blood samples and oral exfoliated cell samples, proving that both sample types are suitable for use with Guangyin Medical Technology Co., Ltd.'s sequencing reaction universal kit.

四、检测结论

本次实验使用血液样本和口腔脱落细胞样本，检测结果的一致性 100%，证明血液样本和口腔脱落细胞样本均适用于广音医疗科技有限公司测序反应通用试剂盒。

济南广音医疗科技有限公司
技术服务部
