# Supplementary material for: OPRM1 gene polymorphism linked to anxiety in cancer-related pain patients: an observational study
Source: Front Pain Res (Lausanne). 2026 Feb 5;7:1666510. doi: 10.3389/fpain.2026.1666510 (PMC12916677; doi:10.3389/fpain.2026.1666510)
Supplement: Supplementary file 5 [file Datasheet2.pdf]

血液和口腔脱落细胞对比验证报告

一 、验证目的

验证血液样本和口腔脱落细胞样本检测结果的一致性，证明血液样本和口腔脱落细胞样本都适用于荧光原位杂交法。验证方法及验证报告如下：

二、验证内容与方法

1. 对象

1.1 测序反应通用试剂盒（鲁济械备 20180020 号）

1.2 样本萃取液（鲁济械备 20180049 号）

1.3 样本：EDTA 抗凝外周血样和口腔拭子各 3 人份（相同 3 人），不分病种、检测目的、性别、年龄。

1.4 检测位点：

MTHFR 068rs1801133(C>T)、ALDH2 020rs671(G>A)、APOE 316rs429358(T>C)、APOE 312rs7412(C>T)、PAI-1 027rs1799762(4G/5G)、MTRR 040rs1801394(A>G)

2. 内容与方法

使用样本萃取液分别处理血液和口腔拭子样本，得到白细胞样本和口腔脱落细胞样本。使用广音医疗科技有限公司的测序反应通用试剂盒进行上机检测，评价如下性能：

结果一致性：将处理后的血液白细胞样本和口腔脱落细胞样本分别加入到 6 位点试剂中，同时上机运行，实验结束后两种样本结果进行比对，得出一致性 100%。

3 实验要求

3.1 实验室严格按照临检中心要求进行分区，保证实验的规范操作和结果的准确可靠。

3.2 实验操作人员应熟悉方法原理与操作，能对样本进行正确处理，并按流程顺利进行白细胞提取、加样、上机。

3.3 实验前，本实验室需按照待评价试剂说明书要求建立实验检测条件。

4 操作程序

4.1 检测流程：EDTA 抗凝血管、口腔拭子管→签收标本→检测前准备→前处理→加样→上机检测→输出结果。

4.2 检测步骤严格按照相关操作规程（SOP）。

三、 验证结果

1 一致性：结果见下表。

孔编号	孔位	位点名称	检测结果	样本编号	病历号	送检日期
0	A1	068rs1801133(C>T)	CT	1	血液	2023-01-10
1	A2	020rs671(G>A)	AG	1	血液	2023-01-10
2	A3	316rs429358(T>C)	TT	1	血液	2023-01-10
3	A4	312rs7412(C>T)	CC	1	血液	2023-01-10
4	A5	027rs1799762(4G/5G)	4G4G	1	血液	2023-01-10
5	A6	040rs1801394(A>G)	AG	1	血液	2023-01-10
6	B1	068rs1801133(C>T)	CC	2	血液	2023-01-10
7	B2	020rs671(G>A)	AG	2	血液	2023-01-10
8	B3	316rs429358(T>C)	TT	2	血液	2023-01-10
9	B4	312rs7412(C>T)	CC	2	血液	2023-01-10
10	B5	027rs1799762(4G/5G)	5G5G	2	血液	2023-01-10
11	B6	040rs1801394(A>G)	AG	2	血液	2023-01-10
12	C1	068rs1801133(C>T)	CT	3	血液	2023-01-10
13	C2	020rs671(G>A)	GG	3	血液	2023-01-10
14	C3	316rs429358(T>C)	TT	3	血液	2023-01-10
15	C4	312rs7412(C>T)	CC	3	血液	2023-01-10
16	C5	027rs1799762(4G/5G)	4G5G	3	血液	2023-01-10
17	C6	040rs1801394(A>G)	AG	3	血液	2023-01-10
18	D1					2023-01-10
19	D2					2023-01-10
20	D3					2023-01-10
21	D4					2023-01-10
22	D5					2023-01-10
23	D6					2023-01-10
24	E1	068rs1801133(C>T)	CT	1	口腔	2023-01-10
25	E2	020rs671(G>A)	AG	1	口腔	2023-01-10
26	E3	316rs429358(T>C)	TT	1	口腔	2023-01-10
27	E4	312rs7412(C>T)	CC	1	口腔	2023-01-10
28	E5	027rs1799762(4G/5G)	4G4G	1	口腔	2023-01-10
29	E6	040rs1801394(A>G)	AG	1	口腔	2023-01-10
30	F1	068rs1801133(C>T)	CC	2	口腔	2023-01-10
31	F2	020rs671(G>A)	AG	2	口腔	2023-01-10
32	F3	316rs429358(T>C)	TT	2	口腔	2023-01-10

33	F4	312rs7412(C>T)	CC	2	口腔	2023-01-10
34	F5	027rs1799762(4G/5G)	5G5G	2	口腔	2023-01-10
35	F6	040rs1801394(A>G)	AG	2	口腔	2023-01-10
36	G1	068rs1801133(C>T)	CT	3	口腔	2023-01-10
37	G2	020rs671(G>A)	GG	3	口腔	2023-01-10
38	G3	316rs429358(T>C)	TT	3	口腔	2023-01-10
39	G4	312rs7412(C>T)	CC	3	口腔	2023-01-10
40	G5	027rs1799762(4G/5G)	4G5G	3	口腔	2023-01-10
41	G6	040rs1801394(A>G)	AG	3	口腔	2023-01-10

2、原始结果截图如下：

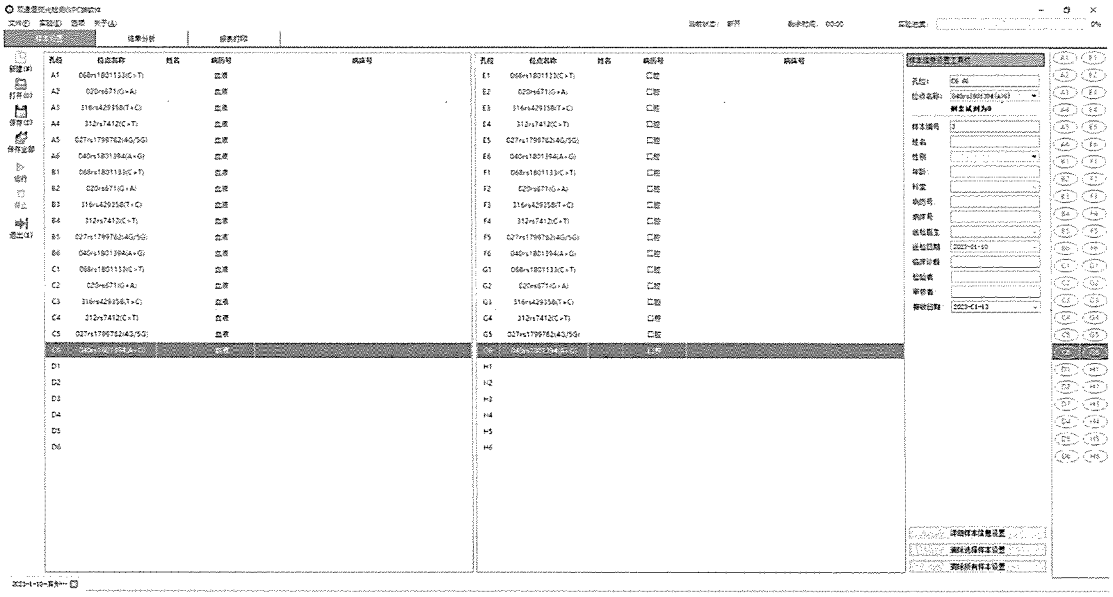

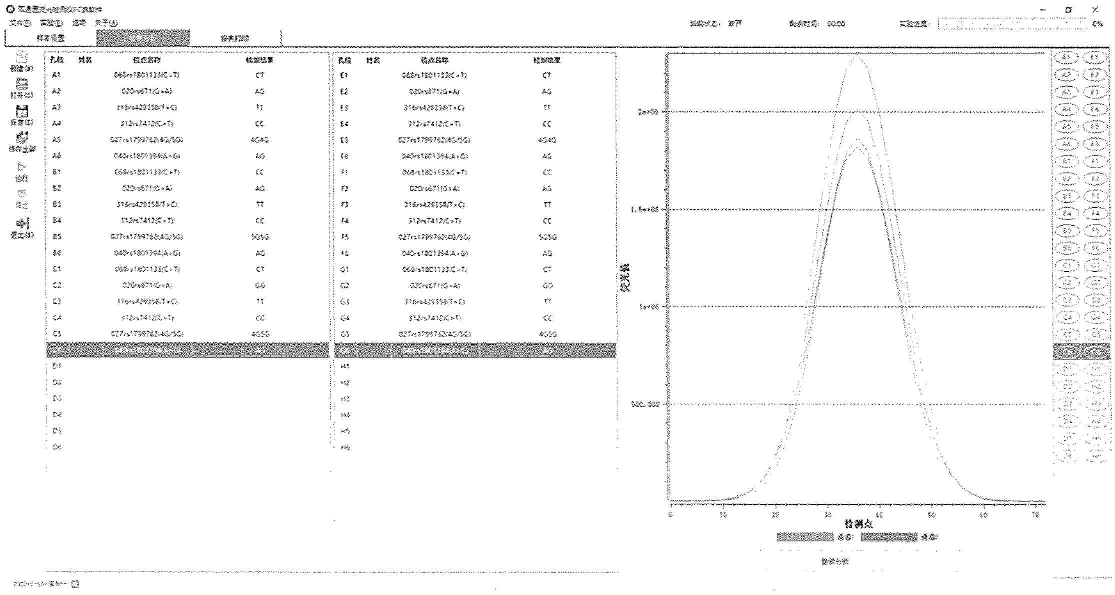

四、检测结论

本次实验使用血液样本和口腔脱落细胞样本，检测结果的一致性 100%，证明血液样本和口腔脱落细胞样本均适用于广音医疗科技有限公司测序反应通用试剂盒。
